# Acute-onset chronic inflammatory demyelinating polyneuropathy following AstraZeneca COVID-19 vaccine: a case report

**DOI:** 10.11604/pamj.2024.47.46.42455

**Published:** 2024-02-06

**Authors:** Emna Smaoui, Khadija Sonda Moalla, Nadia Bouattour, Nouha Farhat, Salma Sakka, Sawsan Daoud, Mariem Damak, Chokri Mhiri

**Affiliations:** 1 Neurology Department, Habib Bourguiba Hospital, Sfax, Tunisia

**Keywords:** Chronic inflammatory demyelinating polyneuropathy, AstraZeneca, COVID-19 vaccine, case report

## Abstract

COVID-19 vaccination side effects have been increasingly reported, including new-onset autoimmune diseases such as chronic arthritis, thrombocytopenia, Guillain-Barré syndrome (GBS), and more recently chronic inflammatory demyelinating polyneuropathies (CIDP). Molecular mimicry and vaccine adjuvants appear to be important contributors to immune-mediated neuropathies. However, whether the link between the COVID-19 vaccine and these autoimmune disorders is coincidental or causal remains uncertain. We describe the ever-reported case of acute-onset CIDP following the Oxford/AstraZeneca vaccine in Tunisia. The patient is a 41-year-old man who presented with acute, worsening weakness of the four limbs. The symptoms appeared 15 days after his first dose of the AstraZeneca vaccine. The diagnosis of GBS was initially confirmed according to the clinical features, the albumino-cytological dissociation in the cerebrospinal fluid (CSF), and the electroneuromyography (ENMG) findings. Serum workup for all known infections associated with immune-mediated neuropathy was negative. The patient was treated with plasma exchange without initial improvement followed by aggravation of the symptomatology after an interval of four and a half months. Control ENMG showed signs of CIDP meeting the European Academy of Neurology/Peripheral Nerve Society (EAN/PNS) criteria of 2021. The patient was treated with maintenance intravenous immunoglobulin and oral corticosteroids. Neurological examination 3 months after discharge showed partial improvement. Worldwide, cases of demyelinating polyneuropathies post-COVID-19 vaccination are increasingly reported. The acute onset of CIDP might lead to a misdiagnosis of GBS. Awareness of this complication and distinction from GBS enables early relay with maintenance treatment to prevent relapses and severe complications. Post-COVID neuropathies are found to be more frequently linked to the AstraZeneca vaccine, however, temporal association does not confirm causal association.

## Introduction

Viral infections and vaccines are important triggers in the pathogenesis of immune-mediated polyneuropathies. Since the coronavirus pandemic, these polyneuropathies have become frequently associated with severe SARS-CoV-2 respiratory syndromes. Moreover, there have been reported cases of immune-mediated polyneuropathies following vaccination against COVID-19, even in the absence of a previous COVID-19 infection. Among these post-vaccination polyneuropathies, chronic inflammatory demyelinating polyneuropathy (CIDP) is relatively rare but is increasingly reported. They can be misdiagnosed as Guillain Barre Syndrome (GBS) in cases of acute onset inflammatory demyelinating polyneuropathies subsequently followed by a relapsing or a progressive course. About 1.5% to 11% of patients with CIDP reported a preceding vaccination within two months from disease onset, which can trigger de novo symptoms or a relapse of CIDP [1]. Herein, we describe an acute-onset inflammatory demyelinating polyneuropathy (A-CIDP), fulfilling the diagnosis criteria of CIDP, in a patient who received the AstraZeneca vaccine, along with a review of the literature. We report this case to increase awareness of A-CIDP as a possible side effect of the COVID-19 vaccine in addition to the GBS, therefore to adapt treatment and prevent relapses.

## Patient and observation

**Patient information:** a 41-year-old man, with no personal history, presented with acute worsening weakness of the lower extremities and paresthesia of the four limbs. There was no history of recent fever, nor signs of gastrointestinal or upper respiratory tract infection. The patient denied any substance or alcohol use and he had no previous SARS-CoV-2 exposure. He had no specific familial medical history, nor are there any noticed similar cases.

**Clinical findings:** neurological examination showed a flaccid tetraparesis predominating in the lower limbs, with areflexia. Motor deficit according to the Medical Research Council (MRC) score was 4/5 in the upper limbs and varied from 1/5 to 3/5 in his lower limbs. He had distal sensory loss and proprioceptive ataxia. Examination of the cranial nerves was normal. No bowel or bladder dysfunction nor respiratory deficiency was reported. His blood pressure was 125/70 mm Hg and his body temperature was 37°C.

**Timeline of current episode:** the patient presented initially generalized arthralgia followed after 4 days by acute worsening weakness of the bilateral lower extremities with paresthesia of the four limbs. The symptoms appeared 15 days after his first dose of the AstraZeneca COVID-19 vaccine. The diagnosis of GBS was initially retained, and the patient was treated with 2 courses of plasma exchange with slight improvement. After four and a half months, the evolution was marked by the aggravation of the paresthesia and weakness of four limbs and the patient became unable to walk even with bilateral assistance. He did not present any respiratory symptoms or cranial nerve palsy.

**Diagnostic assessment:** during the first episode of acute weakness of the lower limbs, the diagnosis of GBS was highly suspected. Routine blood tests including complete blood count were normal. C-reactive protein level was 3.6 mg/dL. Electroneuromyography (ENMG) showed typical features of acute inflammatory demyelinating polyneuropathy (AIDP) meeting the neurophysiological criteria of Doorn *et al*. [2]. Lumbar puncture revealed an albino-cytological dissociation with 4.9 g/L of proteins, 1 white blood cell count/mm^3^, and normoglucorrachia. Lumbar spine Magnetic resonance imaging (MRI) did not show signs of myelopathy. Extensive infectious and inflammatory workup of serum and CSF including HIV antibodies, hepatitis B and C serologies, Lyme, cytomegalovirus (CMV) and Epstein-Barr virus (EBV) serologies, and anti-nuclear antibodies were all negative. Protein electrophoresis was normal and ganglioside antibodies GM1, GD1a, GD1b, GQ1b, and GM2 were negative. The diagnosis of GBS was initially confirmed according to the clinical features, the albumin-cytological dissociation in the CSF, and the ENMG findings. During the relapse: a second lumbar puncture revealed an albumino-cytological dissociation with an increased level of proteins at 6.89 g/L and the control ENMG showed signs of severe CIDP meeting the European Academy of Neurology/Peripheral Nerve Society (EAN/PNS) criteria of 2021.

**Diagnosis:** the diagnosis of acute-onset CIDP was finally made regarding the clinical relapse occurring more than 8 weeks after the first episode and the final results of the control ENMG showing a severe CIDP. The persistence of albumin-cytological dissociation in the CSF further supports this diagnosis.

**Therapeutic interventions:** the patient was then treated with intravenous immunoglobulin (IVIg) at the dose of 0.4g/Kg/day for five days, followed by oral corticoid therapy at the dose of 1mg/Kg/day with progressive tapering. He also received four courses of IVIg along with continuous rehabilitation.

**Follow-up and outcome of interventions:** since the third day of IVIg, the patient noted that the paresthesia disappeared and that his strength began to improve. Four months after discharge and after four courses of IVIg, Neurological examination, and follow-up ENMG showed partial improvement.

**Patient perspective:** the medical care I underwent for my condition has enabled me to regain the ability to walk. While I continue to require rehabilitation, I am satisfied with the quality of care I received. I did not experience any side effects from the treatments.

**Informed consent:** we have obtained an informed consent of our patient for his case to be anonymously published.

## Discussion

The relationship between vaccines and inflammatory neuropathies has been a matter of concern since cases of GBS were discovered following the swine influenza vaccine [3]. Accordingly, cases of GBS and more recently of CIDP have been reported following COVID-19 vaccination. Although GBS was officially recognized as a complication of COVID-19 vaccines, the causal relationship between the new occurrence of CIDP and vaccination against COVID-19 is not yet clear. The Oxford-AstraZeneca COVID-19 vaccine is a viral vector vaccine containing a modified adenovirus ChAdOx1 carrying the coding sequence of the SARS-CoV-2 spike protein. The mechanisms of post-vaccination inflammatory neuropathies may relate to the production of antibodies cross-reacting with neural components, leading to autoimmune destruction of the myelin sheath and/or axonal damage [1,4]. This may involve the SARS-CoV-2 spike protein [5]. It is also possible that the immune target may be related to the adenovirus vector, which may explain the rarity of cases after mRNA vaccines. Other mechanisms include triggering self-reactive T-cell clones, and cytokine upregulation that may induce aberrant MHC class II expression [4,6].

A recent review of the English National Immunization Database of COVID-19 vaccination, found an increased risk of hospitalization and even death, due to GBS occurring up to 28 days following the first dose of the AstraZeneca vaccine [7]. To date, fewer studies have evaluated the association between COVID-19 vaccines and chronic inflammatory neuropathies, but case reports are increasingly published. CIDP is a common acquired autoimmune polyneuropathy characterized typically by a subacute to chronic onset. Acute-onset CIDP (A-CIDP) could occur in 13 to 16% of the cases with initial presentation overlapping clinical and electrophysiological findings with GBS, but subsequently followed by a chronic course beyond eight weeks [8]. Patients with three or more treatment-related fluctuations (TRFs) or presenting clinical deterioration after 8 weeks from disease onset similar to our case are also included in this definition [8].

Chronic inflammatory demyelinating polyneuropathy (CIDP) developing in the post-vaccination period is unusual, estimated at 1.5% to 11% of patients [4]. At the time of writing this paper, 19 cases of post-COVID-19 vaccination new onset CIDP or exacerbation of pre-existing CIDP were reported. Thirteen of them were diagnosed as A-CIDP. Loo and colleagues conducted a study in two Neuroscience centers in the United Kingdom and reported 4 among 16 cases (25%) of acute-onset polyradiculoneuropathy post-COVID-19 vaccination subsequently diagnosed as CIDP. These 4 cases were re-treated with IVIg in two, corticosteroids in one, and plasma exchanges in two with satisfying results [5]. Among the remaining patients, 6 cases occurred after the AstraZenica vaccine, 1 after the Moderna vaccine [6], 1 after inactivated coronavirus vaccine [9], and 1 after Ad26.COV-2. S vaccine [10]. These acute onset CIDP cases are detailed in Table 1, Table 1.1, Table 1.2. All cases but one occurred after the first dose of the received vaccine, the remaining case occurring after the second dose. None of the cases had a history of COVID-19 infection. The average interval between vaccine injection and symptom onset was 25.3 days. Five of these 6 cases are associated with bifacial palsy, unlike our patient. Interestingly, studies have found that post-COVID vaccination acute onset polyneuropathies cases are more commonly associated with cranial nerve involvement, without differences in rates of intensive care unit admission, or ventilator support [5,11]. Treatment consisted of intravenous immunoglobulin (IVIg), plasma exchange, or pulse corticoid therapy for relapses. Maintenance IVIG and/or oral corticoid relay were associated in some cases with Azathioprine or Rituximab as maintenance therapy. The prognosis was favorable in most of the cases. Wen *et al*. reported a case of acute-onset symptoms mimicking typical GBS starting one day after the inactivated COVID-19 vaccine, followed by clinical deterioration 8 weeks later. Detection of NF-186 antibody was positive and the diagnosis of NF186+ CIDP was confirmed [9].

**Table 1:** review of the literature: cases of acute-onset CIDP following COVID-19 vaccination

Publication/country/year	Bagella *et al*./Italy/2021	Suri *et al*./India/2021	Kim *et al*./Republic of Korea/2023
**No of cases**	1	1	1
**Sex/age (years old)**	Man/49	Man/47	Man/72
**Vaccine**	First dose of AZ vaccine	First dose of AZ vaccine	Two doses of tBNT162b2 vaccine; first dose mRNA- 1273 vaccine
**Interval vaccine symptoms (days)**	16	17	30
**Onset (days)**	-	2	15
**Clinical examination**	Bifacial palsy; lower limb areflexia	Severe flaccid quadriparesis; bifacial palsy; right sixth nerve palsy	Distal dominant limb weakness; areflexia; distal sensory deficit in 4 limbs
**ENMG**	Face: reduced CMAP; limbs: sensorimotor D-PNP of the four limbs	D-PNP; abnormal facial and blink reflexes	D-PNP
**CSF**	ACD; (proteins: 110 mg/dL cells<5/mm^3^)	ACD	ACD; (proteins= 72 mg/dL cell < 5 mm^3^)
**Relapses**	At 8 weeks	At 3 weeks; at 8 weeks	At 10 weeks
**Treatment**	Maintenance IVIg	Maintenance IVIg; prednisolone; azathioprine	IVIg; oral prednisolone (30 mg/day); azathioprine
**Evolution**	Improvement	Recovery with no further relapses	Improvement; persistence of mild gait disturbance

AZ: astraZeneca, ENMG: electroneuromyography, CSF: cerebrospinal fluid, ACD: albumino-cytologic dissociation, IVIg: intravenous Immunoglobulin, D-PNP: demyelinating polyneuropathy, CIPD: chronic inflammatory demyelinating polyneuropathy

**Table 1.1:** review of the literature: cases of acute-onset CIDP following COVID-19 vaccination

Publication/country/year	Souza *et al*./Australia/2022		
**No of cases**	3		
**Sex/age (years old)**	Man/51	Man/72	Man/72
**Vaccine**	First dose; AZ vaccine	First dose; AZ vaccine	First dose; AZ vaccine
**Interval vaccine- symptoms (days)**	15	90	15
**Onset (days)**	8	15	8
**Clinical examination**	Bifacial palsy severe quadriparesis; mild sensory loss; dysphagia; dysphonia; respiratory weakness; necessitating MV	Right homonymous hemianopia; paraparesis; hyporeflexia sensory loss	Quadriparesis areflexia; bifacial weakness; right abducent nerve palsy; dysarthria; dysphagia
**ENMG**		Mixed axonal and D-PNP	
**CSF**	ACD (proteins = 0.70 g/L, one lymphocyte).	ACD; (proteins = 2.02 g/L without pleocytosis).	ACD; (proteins=1.964 g/L, one lymphocyte)
**Relapses**	At 3 weeks; at 5 weeks; at 9 weeks	No	at 2 weeks; at 12 weeks
**Treatment**	IVIg; PLEX; maintenance IVIg every 4 weeks	IVIg 2 g/kg; IVIg 0.6 g/kg monthly	IVIg 2 g/kg; IVIg 1 g/kg monthly
**Evolution**	Ambulant with a single-point cane; mild hand weakness persists at 8 months	Improvement in sensory deficits and balance at 8 months	Ambulant with a four-wheeled walker at 5 months

AZ: AstraZeneca, ENMG: electroneuromyography, CSF: cerebrospinal fluid, ACD: albumino-cytologic dissociation, IVIg: intravenous immunoglobulin, PLEX: plasma exchange sessions, MV: mechanical ventilation, PNP: polyneuropathy, CIPD: chronic inflammatory demyelinating polyneuropathy

**Table 1.2:** review of the literature: cases of acute-onset CIDP following COVID-19 vaccination

Publication/country/year	Fotiadou *et al*./Egypt/2022	Wen *et al*./Italy/2022	Our case/Tunisia/2022
**No of cases**	1	1	1
**Sex/age (years old)**	Man/62	Man/23	Man/41
**Vaccine**	First dose; Ad26.COV 2.S vaccine	Second dose; inactivated vaccine	First dose; AZ vaccine
**Interval vaccine- symptoms (days)**	19	29 after the first dose; 1 after the second dose	15
**Onset (days)**	8	--	3
**Clinical examination**	Mild distal weakness; absent achilles tendon reflexes; sensory loss; facial diplegia	Quadriparesis; areflexia; distal sensory deficit; during the relapse: respiratory failure necessitating tracheotomy and mechanical ventilation	Distal dominant quadriparesis; distal sensory loss; proprioceptive ataxia
**ENMG**	Severe bilateral neuropathy with acute and chronic denervation changes	Sensori-motor D-PNP	Sensori-motor D-PNP
**CSF**	ACD; (proteins= 64 mg/dL cells=0)	ACD; (proteins =0.99 g/L)	ACD; (proteins =4.9 g/L, 1 lymphocyte)
**Relapses**	At 58 days	Gradual aggravation during initial treatment; at 8 weeks	At 18 weeks
**Treatment**	IVIG; 7 PLEX sessions; pulsed corticosteroid therapy with oral relay for 4 days every 4 weeks	IVIg; methyl-prednisolone pulse therapy followed by oral relay; PLEX; rituximab	PLEX; IVIG; oral prednisolone with progressive tapering
**Evolution**	Remarkable recovery on 1-month follow-up (GBS-DS=1); facial diplegia persisted	Tardive improvement	Partial improvement

Abbreviations: AZ: astraZeneca, ENMG: electroneuromyography, CSF: cerebrospinal fluid, ACD: albumino- cytologic dissociation, IVIg: intravenous immunoglobulin, PLEX: plasma exchange sessions, D-PNP: demyelinating polyneuropathy, GBS-DS: GBS disability scale, CIPD: chronic inflammatory demyelinating polyneuropathy

We found that most cases are reported after AstraZeneca vaccine, which could be explained by the nature of the vaccine being an adenovirus vector. This case report contributes to the limited cases of CIDP reported after vaccination supporting the hypothesis that vaccination may contribute to the etiology of CIDP. However, temporal association does not imply causality. Our case emphasizes the importance of prolonged follow-up of acute-onset neuropathies for a rapid diagnosis of CIDP to indicate maintenance treatment.

## Conclusion

Vaccines have undoubtedly participated in controlling the COVID-19 pandemic and the important role of COVID vaccines clearly needs emphasizing. Recently, cases of immune mediated neuropathies including CIDP are more and more reported after COVID-19 vaccines. Awareness of this complication and distinction from GBS enables relay with maintenance treatment to prevent relapses. As far as we know, this is the first reported case of acute onset CIDP after AstraZeneca vaccine in Tunisia. Our report highlights the possible relationship between vaccination and the development of CIDP, but definitive confirmation of a causal link needs larger studies.

## References

[1] Singh S, Sanna F, Adhikari R, Akella R, Gangu K (2023). Chronic Inflammatory Demyelinating Polyneuropathy Post-mRNA-1273 Vaccination. Cureus.

[2] van Doorn PA, Van den Bergh PYK, Hadden RDM, Avau B, Vankrunkelsven P, Attarian S (2023). European Academy of Neurology/Peripheral Nerve Society Guideline on diagnosis and treatment of Guillain-Barré syndrome. Eur J Neurol.

[3] Schonberger LB, Bregman DJ, Sullivan-Bolyai JZ, Keenlyside RA, Ziegler DW, Retailliau HF (1979). Guillain-Barre syndrome following vaccination in the National Influenza Immunization Program, United States, 1976--1977. Am J Epidemiol.

[4] de Souza A, Oo WM, Giri P (2022). Inflammatory demyelinating polyneuropathy after the ChAdOx1 nCoV-19 vaccine may follow a chronic course. J Neurol Sci.

[5] Loo LK, Salim O, Liang D, Goel A, Sumangala S, Gowda AS (2022). Acute-onset polyradiculoneuropathy after SARS-CoV2 vaccine in the West and North Midlands, United Kingdom. Muscle Nerve.

[6] Kim S, Lee EK, Sohn E (2023). Two Case Reports of Chronic Inflammatory Demyelinating Polyneuropathy After COVID-19 Vaccination. J Korean Med Sci.

[7] Patone M, Handunnetthi L, Saatci D, Pan J, Katikireddi SV, Razvi S (2021). Neurological complications after first dose of COVID-19 vaccines and SARS-CoV-2 infection. Nat Med.

[8] Suri V, Pandey S, Singh J, Jena A (2021). Acute-onset chronic inflammatory demyelinating polyneuropathy after COVID-19 infection and subsequent ChAdOx1 nCoV-19 vaccination. BMJ Case Rep.

[9] Wen S, Huang K, Zhu H, Li P, Zhou L, Feng L (2022). Case Report: Anti-NF186+ CIDP After Receiving the Inactivated Vaccine for Coronavirus Disease (COVID-19). Front Neurol.

[10] Fotiadou A, Tsiptsios D, Karatzetzou S, Kitmeridou S, Iliopoulos I (2022). Acute-onset chronic inflammatory demyelinating polyneuropathy complicating SARS-CoV-2 infection and Ad26.COV2.S vaccination: report of two cases. Egypt J Neurol Psychiatr Neurosurg.

[11] Bagella CF, Corda DG, Zara P, Elia AE, Ruiu E, Sechi E (2021). Chronic Inflammatory Demyelinating Polyneuropathy after ChAdOx1 nCoV-19 Vaccination. Vaccines.

